# Opportunities for employers to address the opioid epidemic: results from a national survey

**DOI:** 10.1093/haschl/qxaf155

**Published:** 2025-08-29

**Authors:** Gillian K SteelFisher, Brian C Castrucci, Mary G Findling, Emma C Prus, Jazmyne Sutton, Michael L Barnett

**Affiliations:** Department of Health Policy and Management, Harvard T.H. Chan School of Public Health, Boston, MA, USA; The de Beaumont Foundation, Bethesda, MD, USA; Department of Health Policy and Management, Harvard T.H. Chan School of Public Health, Boston, MA, USA; The de Beaumont Foundation, Bethesda, MD, USA; SSRS, Glen Mills, PA, USA; Department of Health Policy and Management, Harvard T.H. Chan School of Public Health, Boston, MA, USA

**Keywords:** opioid use disorder, opioid epidemic, recovery ready workplace, employees, employers, public opinion, survey, Narcan, naloxone

## Abstract

**Introduction:**

Employers play a critical role in addressing the opioid crisis in the United States, so the federal government created the Recovery-Ready Workplace (RRW) framework, which suggests employer action in 4 areas: prevention and risk reduction, education, employment, and treatment.

**Methods:**

This study examines employees’ views of RRW-related actions taken by their employers to address opioid misuse, using a national survey of 1010 U.S. employees.

**Results:**

Results showed significant shortfalls across RRW areas. Only 19% of employees said Narcan was available at work, and only 27% said their employer offered opioid prevention services. Moreover, 79% believed that most coworkers would not be very comfortable receiving opioid abuse prevention information from their employer. Majorities supported retaining co-workers in opioid treatment when they had strong performance records (72%), were monitored (64%), or never misused opioids during work (61%). However, one-third (33%) believed that if employees with good records sought treatment, their employer would be more likely to look for ways to fire rather than support the employee.

**Conclusion:**

More employer action is needed, with consideration for expanding Narcan availability, increasing employee access to prevention programs, enhancing employment practices to keep people in the workforce, and cultural shifts to reduce stigma.

## Teaser

The opioid epidemic is one of the most urgent health issues in the United States. Given its complex roots and pervasive impact, a multisectoral approach is needed to address it, and increasingly, attention is being paid to the role of employers and businesses as an important sector for addressing this crisis. To guide employers’ efforts, the federal government created the Recovery-Ready Workplace (RRW) framework, which suggests employer action in four areas: prevention and risk reduction, education, employment, and treatment. This survey study provides an assessment of workplace readiness by examining employees’ views of RRW-related actions taken by their employers, intended to guide employers’ future efforts.

## Introduction

The opioid epidemic is one of the most urgent health issues in the United States. Given its complex roots and pervasive impact, a multisectoral approach is needed to lower rates of opioid misuse and opioid use disorder (OUD).^1-4^ Increasingly, employers and businesses have been recognized as an important sector for addressing this crisis, as approximately half of the US adults who misuse opioids are employed, and most employers say opioid use has impacted their workplace, yet few believe they are very well prepared to address it.^1-7^ Further, workplace factors, such as labor-intensive occupations and overprescribing after workplace injuries, are known contributors.^1-3,8,9^

To help employers prevent and respond more effectively to opioid misuse among their workforce, the federal government created an interagency working group to develop the Recovery-Ready Workplace (RRW) framework.^5^ This framework was created as one of the top priorities of the Biden Administration's $40 billion National Drug Control Strategy in 2022.^10^

The RRW framework includes 4 pillars for employer action: OUD prevention and risk reduction; training and education; hiring and employment; and treatment and recovery support (see Supplementary material for details).

Despite such intense investment in RRW, no studies have examined the extent of employer actions on RRWs. This study fills this gap by assessing employees’ views of RRW-related actions taken by employers. Surveying employees directly provides insight by measuring how employers’ actions are experienced by the employees who need them.

## Data and methods

### Survey design and population

Data come from a nationally representative, probability-based survey sample of 1010 US employees, defined as adults aged 18 years and older, who reported being employed at companies with 50+ employees. Those at companies with <50 employees were excluded, as they are often exempt from federal requirements like offering health insurance, affecting access to addiction care. The study was determined to be exempt by the Harvard T.H. Chan School of Public Health Institutional Review Board. The Supplementary material contains additional methodological details.

### Data collection

Data were collected from October 2 to 16, 2024, via online and telephone surveys using the SSRS Opinion Panel, a high-quality, probability-based panel designed to be representative of the US adult population.^11^ Panelists were recruited using methods allowing for randomized selection from the broader US population: by mail (address-based sampling) and phone (random digit dialing). Panelists were screened for eligibility at the beginning of the survey and given small monetary incentives for completion.

### Survey instrument and measures

This study followed American Association of Public Opinion Research best practices and reporting guidelines.^12^ The research team created the questionnaire after reviewing the RRW toolkit and prior qualitative and quantitative research on workplace opioid use, prevention, and treatment.^2,4,6,7,13-16^ They used cognitive interviews to revise the questionnaire by improving comprehension and minimizing bias.

The survey instrument asked participants about their views of their employers’ actions with respect to the 4 RRW pillars. Participants also self-reported demographic and employment characteristics.

### Statistical analysis

Key demographic variables were weighted to national benchmarks to reflect the employed US population at companies with 50+ employees (not self-employed). See Supplementary material for detailed weighting procedures.

We examined overall responses and differences in responses across employer size and manual labor roles. Employer size was categorized based on number of employees: smaller (50-<100 employees), medium (100-<1500 employees), and larger (1500+ employees). We used 2-tailed *t-*tests to compare bivariate differences; only statistically significant results (*P* < .05) are reported in the results. All analyses were performed in Stata, version 15.0, with weighted data.

### Limitations

Limitations of this study include those common in survey research. Data are self-reported, and there is a potential for nonresponse bias beyond what can be addressed with weighting. Data are cross-sectional and should not be interpreted as causal. Views may change over time. We only examined employees’ perspectives and may thus under-report the extent of employers’ actions if employees were not aware of them. Employer actions may also differ among self-employed adults or those working for smaller employers.

## Results

The response rate among those invited to participate in this study, adjusting for estimated eligibility, was 46%. The final sample included 1010 US employees, with sociodemographic characteristics shown in Table 1.

**Table 1.** Sociodemographic characteristics of the weighted sample of the adult US workforce at companies with 50+ employees.

Characteristic	Unweighted *n*	Weighted percent
Gender		
Men	504	52
Women	504	48
Age		
18-24	58	8
25-34	272	25
35-44	242	24
45-54	203	22
55-64	185	18
65+	50	4
Race and ethnicity		
White, non-Hispanic	574	62
Black, non-Hispanic	172	13
Hispanic/Latino	185	16
Asian, non-Hispanic	79	9
Metropolitan status		
Urban	341	31
Suburban	556	57
Rural	100	10
Education		
HS grad or less	307	30
Some college	266	22
College+	437	48
Manual labor job		
Yes	521	51
No	489	49
Employer size		
50-<100 employees	180	18
100-1499 employees	318	31
1500+ employees	512	51
Employment status		
Full-time	881	86
Part-time	129	14

Data obtained from an October 2024 nationally representative survey of 1010 employed US adults. Weighted percentages and unweighted sample sizes are displayed. Percentages may not add up to 100% due to rounding or do not know/missing responses. Additional details on weighting and methods are available in the Supplementary material.

### Preventing opioid abuse and lowering acute overdose risk

Only about 1 in 4 employees (27%) said their employer offered programs or services to help prevent opioid abuse, while 23% said their employer did not, and half (50%) did not know. One in 5 (19%) said their employer offered Narcan on-site, while one-third (34%) said their employer did not, and 47% said they did not know. Among employees who did not indicate that their employer offered Narcan on-site, nearly two-thirds (63%) said they believe their employer *should* do this (Table 2).

**Table 2.** US workforce perspectives on their employers’ actions on opioid use disorder related to core recovery-ready workplace pillars (weighted percent).

	Total	Manual labor job		Employer size (No. employees)		
**Pillar I: views on preventing opioid abuse and lowering acute overdose risk**						
Employer offers programs/services to help prevent opioid abuse among employees		Yes (*n* = 521)	No (*n* = 489)	<100 (*n* = 180)	100-1499 (*n* = 533)	1500+ (*n* = 297)
Yes	27	30	24	19	27	30^c^
No	23	22	24	33^e^	30^e^	16
Don’t know	50	48	52	48	43	54^d^
Employer offers Narcan on-site for employees, customers, or others who might need it						
Yes	19	23^b^	16	19	23	17
No	34	37	30	45	38	27
Don’t know	47	40	54^a^	36	39	55
Employer should make Narcan available on-site^f^						
Yes	63	65	61	58	63	65
No	37	35	39	42	37	35
**Pillar II: views on education and training**						
Employer should provide employees with general information about the risks of opioid abuse						
As a standard practice	51	54	47	41	52	53^c^
Only if employees seek it out	38	37	38	42	34	39
Not at all	12	9	15^a^	17	15	8
Employer should provide employees with guidance after injuries when employees may be prescribed opioids for pain						
As a standard practice	52	54	49	40	52^c^	56^c^
Only if employees seek it out	37	36	38	43	36	36
Not at all	11	9	13	16^e^	13	8
Employer should provide employees with information about keeping prescription medication away from family members						
As a standard practice	43	46	40	39	41	46
Only if employees seek it out	42	42	42	42	43	42
Not at all	15	12	17	19	17	12
How comfortable most coworkers would be getting opioid abuse prevention information from their employer						
Very comfortable	22	24	19	21	22	22
Somewhat comfortable	51	49	52	48	49	55
Not too comfortable	21	19	22	22	24	15
Not comfortable at all	7	8	6	9	6	8
Reasons employees think most of their coworkers would not be very comfortable getting this information from their employer^g^						
They would be worried about privacy	73	69	76^a^	67	77	72
They would be worried that they would be treated differently	68	64	73^a^	64	70	69
They would be worried that it would raise suspicion from their coworkers or supervisors	67	64	70	58	74^c^	66
They would be worried that my employer had ulterior motives	47	47	46	44	51	45
They would not trust the quality of information provided by my employer	29	32	27	32	33	26
**Pillar III: views on hiring and employment**						
Employers should keep current employees who are in recovery from opioid addiction and receiving treatment under each circumstance						
They have done a good job at work	72	72	72	68	76	71
They would be required to undergo regular performance monitoring by employer	64	66	62	65	66	62
They have never misused opioids during work	61	60	63	61	62	61
They have contact with customers	52	53	51	57	54	49
They have teaching or training responsibilities	49	50	48	49	53	47
They have frontline care responsibilities (eg, nurse)^h^	43	—	—	—	—	—
They have managerial responsibilities	43	41	46	48	46	40
Employer should provide employees in recovery with flexible schedules to receive treatment, as long as the work can be done that way^i^						
Yes	79	78	80	74	76	83
No	9	10	8	11	8	8
Don’t know	12	12	13	16	16	9
**Pillar IV: views on treatment and recovery support**						
Employer offers health insurance that covers treatment for employees’ opioid addiction						
Yes	23	21	26	22	20	26
No	16	21	11	23	18	12
Don’t know	61	58	64	55	61	63
Anticipated reaction of your employer to an employee who has a good record at work, but wants treatment for opioid addiction						
Would be more likely to look for ways to support the employee through treatment	67	63	72^a^	62	67	69
Would be more likely to look for ways to fire the employee	33	37^b^	28	38	33	31

Weighted percentages and unweighted sample sizes are displayed. Percentages may not add up to 100% due to rounding or do not know/missing responses.

^a^Significantly higher than those in manual labor jobs at *P* < .05.

^b^Significantly higher than those in non-manual labor jobs at *P* < .05.

^c^Significantly higher than those working for employers with <100 employees at *P* < .05.

^d^Significantly higher than those working for employers with 100-1499 employees at *P* < .05.

^e^Significantly higher than those working for employers with 1500+ employees at *P* < .05.

^f^Only asked among the subset (*n* = 824) whose employers do not offer Narcan or are unsure if their employer offers Narcan.

^g^Only asked among the subset (*n* = 776) of those who did not think employees would be “very comfortable” getting opioid abuse prevention information from their employer.

^h^Only asked among those who work in healthcare, *n* = 193, too few cases for subgroup analysis.

^i^Only asked among the subset (*n* = 828) of those who said “yes” to keeping employees in recovery and receiving treatment under any of the listed circumstances.

### Education and training

Most (89%) said their employer should provide employees with general information about opioid abuse risks, either as a standard practice (51%) or if employees seek it out (38%; Table 2). Similarly, most said their employer should provide employees with guidance after injuries when employees may be prescribed opioids for pain (89%) and should provide employees with information about keeping prescriptions away from family members (85%).

However, there were social barriers to information uptake, as the vast majority (79%) indicated they did not think most of their coworkers would be “very comfortable” getting opioid abuse prevention information from their employer. Reasons employees thought coworkers would be uncomfortable included concerns about privacy (73%), being treated differently (68%), or raising suspicion from coworkers or supervisors (67%). Nearly half said they would be worried their employer had ulterior motives (47%).

### Hiring and employment

Majorities of employees supported retaining coworkers in treatment for opioid abuse if employees had done a good job at work (72%), were monitored by their employer (64%), or had never misused opioids during work (61%; Table 2). Support for retaining coworkers in treatment declined if employees had contact with customers (52%), teaching and training responsibilities (49%), or managerial responsibilities (43%). Among employees in healthcare, only 43% supported retaining coworkers in treatment if they had frontline care responsibilities.

Most of those who supported keeping employees in recovery and receiving treatment under any of the listed circumstances (79%) said their employer should provide employees in recovery with flexible schedules and treatment, as long as the work can be done that way.

### Treatment and recovery support

The majority of employees (61%) did not know if their employer offered health insurance covering treatment for opioid addiction, while 23% said their employer did offer this, and 16% said they did not (Table 2). We also found sizeable stigma related to seeking treatment, as one-third of employees (33%) believed that if employees with good records at work sought treatment for opioid addiction, their employer would be more likely to look for ways to fire the employee than support them.

### Subgroup differences

We found few differences between responses of employees with manual or physical labor jobs vs those in nonlabor jobs. Those with manual labor jobs were slightly more likely to report Narcan being offered at work (23%-16%), though still fewer than 1 in 4 said this. Those with manual labor jobs were also slightly more likely to say their employers should provide employees with general opioid abuse risk information, though support was high in both settings (91%-85%). Lastly, employees with manual labor jobs were more likely to believe coworkers might be fired for seeking opioid addiction treatment (37%-28%).

There were similarly few differences in results by employer size. Employees at larger companies were more likely than those at smaller companies to say their employer offered prevention programs (30%-19%). They were also more likely to say their employer should provide information about opioid abuse risks as standard practice (53%-41%). Employees at larger and medium-sized companies were also more likely than those at smaller companies (56% and 52% versus 40%) to say their employer should provide guidance after injuries when employees may be prescribed opioids.

## Discussion

Results from this national study suggest that most employees do not think their employers are “recovery ready.” In each pillar of action outlined in the RRW framework, there is low employee awareness of, access to, and confidence in relevant information, programs, and services. Moreover, neither companies supporting employees at higher risk, like those with manual labor jobs, nor companies with greater resources, like larger companies, appear to be consistently or meaningfully more likely than their counterparts to be taking relevant actions. Results indicate that substantially more effort is needed across the business sector to help prevent and address the opioid crisis. Moreover, employees support many of these actions, suggesting feasibility and acceptability for enhancing initiatives in the future. In particular, 3 challenges and opportunities stand out.

First, employees have substantial interest in their employers providing both standard information about the risks of opioid misuse to employees and targeted information after injury, as well as interest in having Narcan available at their worksites. Given that most states now have Good Samaritan laws to protect bystanders who respond,^17^ businesses are a potentially reliable source for Narcan to reduce the tragic consequences of overdoses.^16^ Training multiple employees to be able to administer Narcan can make these programs more effective. In turn, supporting public health agencies to provide such training can reduce the burden on businesses and facilitate important cross-sector partnerships.

Second, we found sizable stigma related to seeking treatment in the workplace, with many believing that employers would be looking for ways to fire them even if they were otherwise in good standing. Further, we found stigma even in seeking information about preventing opioid misuse, with many employees saying they would not feel very comfortable getting such information from their employers. Thus, major cultural shifts are needed to reduce stigma and facilitate conversation between employers and employees. Increased training for human resources staff to provide nonstigmatizing support, as well as broader awareness and education campaigns on worksites, may help make this shift.^18^ Ultimately, having more employees in treatment is beneficial not only to the societal good of reducing OUD but also directly to employers, as receiving treatment often improves employees’ productivity.^19^

Finally, results suggest employees are largely supportive of keeping people employed while they receive needed treatment, so long as they are otherwise in good standing. Further, employees support the flexibility often needed for people to get treatment, such as remote work when possible.^5^ This may open opportunities for creating more supportive employment environments, which can help motivate people to enter treatment and adhere to medication regimens,^2,20-22^ while benefiting employers by reducing the costs of hiring and training new staff. To create supportive environments, employers may seek to leverage tax credits or other financial incentives, as well as expand partnerships with organizations that leverage relevant expertise.^5^ Additional local, state, or federal guidance for employers navigating the development of employee supports, including monitoring, placement assistance, and flexibility for accessing treatment, could also be helpful. Finally, beyond the individual employer level, the broader business community may lead multiemployer efforts to offer training, technical assistance, and even certification as RRWs.^5^

To complement these learnings about employees’ views, future research should examine employers’ knowledge, implementation of, and hesitations about RRWs. This would help bridge perspectives, clarifying whether the most effective next steps in enhancing efforts should focus on supporting additional programs and services from employers with education or incentives, or whether the most effective next steps should focus on increasing employee awareness of existing programs and services.

## Conclusion

Results suggest immense opportunity to mobilize employers in efforts to address OUD in the workforce across prevention and acute response, education, employment practices, and treatment. Policies to shape and motivate employers’ responses, such as expanding Narcan availability, increasing employee awareness of prevention and treatment programs, and expanding supportive recovery environments that retain employees while they get treatment, could be critical tools to help address the US opioid crisis. Similarly, more efforts to make employees aware of existing efforts may be helpful. To be successful, cultural shifts to reduce stigma around seeking prevention and treatment are also needed.

## Supplementary Material

qxaf155_Supplementary_Data
