# Supplementary material for: Opportunities for employers to address the opioid epidemic: results from a national survey
Source: Health Aff Sch. 2025 Aug 29;3(8):qxaf155. doi: 10.1093/haschl/qxaf155 (PMC12394939; doi:10.1093/haschl/qxaf155)
Supplement: qxaf155_Supplementary_Data [file qxaf155_supplementary_data.zip › FINAL SUPPLEMENT_2025.pdf]

Supplemental Online Content

Table of Contents

A) Recovery-Ready Workplace (RRW) Pillars 2

B) Methods Supplement: Questionnaire Development and Question Language 3

C) Methods Supplement: Sampling, Weighting, and Margin of Sampling Error Details 5

D) Methods Supplement: Weighting Variables and Sources 6

E) Methods Supplement: Weighting Variables and Comparison Between Weighted Sample and National Benchmarks 7

This supplemental material has been provided by the authors to give readers additional information about their work.

A) Recovery-Ready Workplace (RRW) Pillars

The principles listed below and the related metrics in this survey study were taken from the Federal Recovery-Ready Workplace Interagency Workgroup, *Recovery-Ready Workplace Toolkit: Guidance and Resources for Private and Public Sector Employers*, U.S. Department of Labor; 2023. www.dol.gov/agencies/eta/RRW-hub/Toolkit

**Federal Recovery-Ready Workplace (RRW) Interagency Workgroup**<br>**Summary of RRW Pillars**
**<u>Pillar I: Prevention and Risk Reduction</u>**<br><br>Preventing substance use disorder in the workplace is comprised of employers' actions, policies, and programs to reduce risk factors for substance misuse in the workplace.
**<u>Pillar 2 - Training and Education</u>**<br>**<u>(includes stigma reduction efforts)</u>**<br><br>Training and educating employees include increasing their understanding of substance use disorder and recovery; training on organizational substance use policies and related benefits and resources; reducing organizational stigma; increasing literacy on substance abuse disorder and recovery.
**<u>Pillar 3 - Hiring and Employment</u>**<br><br>Creating a recovery-ready workplace through hiring and employment policies include examining hiring policies; how employers and businesses respond to substance misuse among employees; and return-to-work policies/plans after treatment for substance use disorder.
**<u>Pillar 4 - Treatment and Recovery Support</u>**<br><br>Employers most commonly provide access to treatment and support for recovery through employee assistance programs and health insurance benefits. They may also develop relationships with local treatment providers and others in the community to ensure employees have access to services they need.

B) Methods Supplement: Questionnaire Development and Question Language

Details on Questionnaire Development
The questionnaire was developed using American Association of Public Opinion Research *Best Practices for Survey Research*.[1] Data come from an online and telephone survey using a nationally-representative, probability-based sample of 1,010 US adults, aged 18 years and older, who reported being employed full-time or part-time. Self-employed adults and adults working at companies with fewer than 50 employees were excluded.

QUESTIONS	RESPONSE CATEGORIES
Questions Related to Pillar I: Views on Preventing Opioid Abuse and Lowering Acute Overdose Risk	
As far as you know, does your employer offer any programs or services to help prevent opioid abuse among its employees?	Yes<br>No<br>Don't know
There is an over-the-counter medication called Narcan, also known as Naloxone, that can be given in an emergency and prevent people from dying if they are experiencing an opioid overdose. As far as you know, does your employer offer Narcan on site for employees, customers, or others who might need it?	Yes<br>No<br>Don't know
(ASKED OF THOSE WHOSE EMPLOYERS DO NOT OFFER NARCAN OR ARE UNSURE IF THEY DO, n=824)<br>In your view, should your employer make Narcan available on site for employees, customers, or others who might need it?	Yes<br>No
Questions Related to Pillar II: Views on Education and Training	
Do you think your employer should provide employees with each of the following as standard practice, only if employees seek it out, or not at all?<br>a. General information about the risks of opioid abuse<br>b. Guidance after injuries when employees may be prescribed opioids for pain<br>c. Information about keeping prescription pain medication away from family members	As a standard practice<br>Only if employees seek it out<br>Not at all
If your employer did offer this kind of information, in general, how comfortable do you think most of your coworkers would be getting this information from your employer?	Very comfortable<br>Somewhat comfortable<br>Not too comfortable<br>Not comfortable at all
(ASKED OF THOSE WHO THINK EMPLOYEES WOULD NOT BE VERY COMFORTABLE GETTING INFORMATION FROM EMPLOYER, n=776)<br>Are each of the following a reason you think most of your coworkers would not be very comfortable getting this information from your employer, or is it not a reason?<br>a. They would be worried that it would raise suspicion from their coworkers or supervisors.	Yes, it is a reason<br>No, it is not a reason

[1] American Association for Public Opinion Research (AAPOR). Best Practices for Survey Research [Internet]; 2022. https://www.aapor.org/Standards-Ethics/Best-Practices.aspx#best3

b. They would not trust the quality of information provided by my employer.<br>c. They would be worried that my employer had ulterior motives.<br>d. They would be worried that they would be treated differently<br>e. They would be worried about privacy	
Questions Related to Pillar III: Views on Hiring and Employment	
In your view, should your employer keep current employees who are in recovery from opioid addiction and receiving treatment under the following circumstances?<br>a. Have done a good job at work<br>b. Have never misused opioids during work<br>c. Would be required to undergo regular performance monitoring by my employer<br>d. Have contact with customers<br>e. Have managerial responsibilities<br>f. Have teaching or training responsibilities<br>(only asked among those who work in healthcare, n=193) g. Have frontline care responsibilities (e.g., nurse)	Yes<br>No<br>Don't know
**(ASKED OF THOSE WHO SAID YES TO ANY ITEM IN Q47, n=828)**<br>Do you think employees in recovery from opioid addiction should be offered flexible schedules to receive treatment, as long as the work can be done that way?	Yes<br>No<br>Don't know
Questions Related to Pillar IV: Views on Treatment and Recovery Support	
Does your employer offer you health insurance? And then among those whose employers offer health insurance, follow up question: As far as you know, does that health insurance cover each of the following? a. Treatment for opioid addiction	Yes<br>No<br>Don't know
If you had to guess, which would you say best describes how your employer would react to an employee who has a good record at work, but wants treatment for opioid addiction? Your employer would be…	More likely to look for ways to support the employee through treatment<br>More likely to look for ways to fire the employee
Demographic and Profiling Questions	
How many employees, including full-time and part-time, does your workplace organization have across all locations?	50-99<br>100-499<br>500-1499<br>1500-1999<br>2000 or more
Does your job involve any of the following duties or tasks?<br>a. Manual or physical labor	Yes<br>No

C) Methods Supplement: Sampling, Weighting, and Margin of Sampling Error Details

Respondents were reached through the SSRS Opinion Panel,[2] a nationally representative probability-based web panel. Panelists who previously mentioned being employed at a company with 10+ employees were invited to take the survey. All invited panelists were re-screened for current employment status and the size of the company where they work. Demographic characteristics used in weighting were also asked of all invited panelists. After screening, only panelists who reported currently working at a company with 50+ employees were eligible to take the full survey. Given that this is a probability-based panel, findings are statistically projectable to the general U.S. population of employed adults (self-employed excluded). A total of 962 surveys were completed by panelists online, and 48 interviews were completed by phone with panelists who do not have access to the internet, or who do not use it. The survey was administered from October 2 – October 16, 2024, in both English and Spanish.

The sample was weighted in stages. The 2023 US Census Current Population Survey does not provide estimates for employees at companies of 50+ employees but instead uses categories of <10, 10-24, 25-99, and so on. Other sources for benchmarks also differ in availability of an estimate for those at companies with 50+ employees. Therefore, data were weighted initially to represent the U.S. adult population working full or part time at a company with 10+ employees. The data were weighted by applying a base weight and balancing the demographic profile of the sample to target population parameters. After the data were weighted to that population, the data were filtered and standardized overall to those who work full or part time at a company with 50+ employees. Data were weighted to account for different selection probabilities and response rates across sample strata. Data were then weighted to balance the demographic profile of the sample to selected population parameters. Data were weighted by gender, age, education, race and ethnicity, Census region, home tenure, number of adults per household, population density, religion affiliation, political party, and voter registration. Data were also weighted by additional metrics standard among many internet wave panels to reduce nonresponse bias – internet use and, civic engagement – because civically engaged respondents and higher-frequency internet users differ from the general population and are over-represented among panelists.[3] Weights were trimmed to prevent individual interviews from having too much influence on final results.

Comparisons between the survey sample and national benchmarks for key demographics are shown later in the Supplement. The design effect for this survey was 1.55, and the margin of error for the entire sample is ±3.8 percentage points at the 95% confidence interval.

The participation rate for this survey was expected due to the rapid response nature of fielding the survey. Prior research suggests that the resulting data are comparable to longer-term, higher-response surveys when reweighted to key population parameters.[4]

---

[2] https://ssrs.com/opinion-panel/

[3] See, for example, Amaya and Presser. Nonresponse Bias for Univariate and Multivariate Estimates of Social Activities and Roles. *Public Opin Q*. 2016; Greenberg and Schneider. Population density: What does it really mean in geographical health studies? *Health Place.* 2023;81:103001; American Association for Public Opinion Research (AAPOR). Data Quality Metrics for Online Samples: Considerations for Study Design and Analysis. November 2022. Available online: https://aapor.org/wp-content/uploads/2023/02/Task-Force-Report-FINAL.pdf

[4] See, for example, Kohut et al. Assessing the Representativeness of Public Opinion Surveys. Pew Research Center. 2012. http://www.people-press.org/2012/05/15/assessing-the-representativeness-of-public-opinion-surveys; Mercer A, Lau A. Comparing Two Types of Online Survey Samples. Pew Research Center; 2023. https://www.pewresearch.org/methods/2023/09/07/comparing-two-types-of-online-survey-samples/; Keeter S, Hatley N, Kennedy C, Lau A. What Low Response Rates Mean for Telephone Surveys. Pew Research Center; 2017. https://www.pewresearch.org/wp-content/uploads/2017/05/RDD-Non-response-Full-Report.pdf.

D) Methods Supplement: Weighting Variables and Sources

Variables Weighted:	Weighted to:
Gender<br>Age<br>Education<br>Race<br>Ethnicity/Hispanic nativity<br>Census region<br>Home tenure<br>Number of adults per household	2023 Current Population Survey[5]
Population density	Claritas Pop-Facts Premier 2023[6]
Religion affiliation<br>Internet frequency<br>Party ID	Pew Research Center's National Public Opinion Reference Survey (NPORS)[7]
Civic engagement	September 2021 CPS Volunteering and Civic Life Supplement[8]
Voter registration	CPS 2022 Voting and Registration Supplement[9]

[5] Sarah Flood, Miriam King, Renae Rodgers, Steven Ruggles, J. Robert Warren, Daniel Backman, Annie Chen, Grace Cooper, Stephanie Richards, Megan Schouweiler and Michael Westberry. IPUMS CPS: Version 11.0 [dataset]. Minneapolis, MN: IPUMS, 2023. https://doi.org/10.18128/D030.V11.0

[6] https://environicsanalytics.com/data/demographic/pop-facts-premier

[7] https://www.pewresearch.org/methods/fact-sheet/national-public-opinion-reference-survey-npors/ - Feb 1 to Jun 10, 2024

[8] Sarah Flood, Miriam King, Renae Rodgers, Steven Ruggles, J. Robert Warren and Michael Westberry. Integrated Public Use Microdata Series, Current Population Survey: Version 10.0 [dataset]. Minneapolis, MN: IPUMS, 2022. https://doi.org/10.18128/D030.V10.0

[9] Sarah Flood, Miriam King, Renae Rodgers, Steven Ruggles, J. Robert Warren, Daniel Backman, Annie Chen, Grace Cooper, Stephanie Richards, Megan Schouweiler and Michael Westberry. IPUMS CPS: Version 11.0 [dataset]. Minneapolis, MN: IPUMS, 2023. https://doi.org/10.18128/D030.V11.0

E) Methods Supplement: Weighting Variables and Comparison Between Weighted Sample and National Benchmarks

Variable		National Benchmark among those at companies with 10+ employees %	Weighted Sample of those at companies with 10+ employees %	Weighted sample of those at companies with 50+ employees %
Gender	Men	52.1%	52.0%	52.1%
	Women	47.9%	48.0%	47.9%
Age (in years)	18-24	11.4%	10.9%	8.2%
	25-34	24.0%	23.8%	24.7%
	35-44	22.7%	22.9%	24.2%
	45-54	20.2%	20.3%	21.9%
	55-64	16.2%	16.4%	17.5%
	65+	5.6%	5.7%	3.5%
Education	≤HS	30.5%	30.4%	29.9%
	Some College	25.9%	25.8%	21.6%
	College+	43.6%	43.8%	48.5%
Region	Northeast	17.4%	17.6%	16.5%
	Midwest	21.6%	21.7%	23.6%
	South	37.5%	37.4%	37.5%
	West	23.5%	23.3%	22.4%
Civic Engagement	Engaged	30.9%	30.7%	33.0%
	Not Engaged	69.1%	69.3%	67.0%
Race and Ethnicity	White, Non-Hispanic	59.9%	60.4%	62.1%
	Black, Non-Hispanic	12.7%	12.9%	12.6%
	Hispanic/Latino	17.7%	17.3%	16.4%
	Asian, non-Hispanic	6.9%	6.9%	6.2%
	Other, non-Hispanic	2.8%	2.5%	2.6%
Density Quintiles	1	17.1%	17.4%	16.6%
	2	19.6%	19.5%	18.8%
	3	20.6%	20.5%	21.3%
	4	22.2%	22.1%	22.8%
	5	20.6%	20.4%	20.5%
Internet Use Frequency	Almost constantly	55.0%	55.2%	54.1%
	Several times a day	40.7%	40.4%	42.0%
	Less often/Not a user	4.3%	4.4%	3.9%
Registered Voter	Yes	80.4%	80.8%	82.7%
	Not registered/ no response	19.6%	19.2%	17.3%
Party ID	Rep	26.5%	26.7%	26.2%
	Dem	30.3%	30.3%	31.9%
	Ind/Other	43.3%	43.0%	41.9%
Religious Affiliation	Affiliated	66.9%	66.7%	65.7%
	Not affiliated	33.1%	33.3%	34.3%
Number of Adults in HH	1 adult	15.0%	15.4%	16.4%
	2 adults	54.4%	54.4%	54.6%
	3+ adults	30.6%	30.3%	29.0%
Home Tenure	Own	67.9%	67.6%	68.6%
	Rent	32.1%	32.4%	31.4%
